# Two Cases Diagnosed With Wallenberg Syndrome After Developing Dysphagia Following COVID-19 Infection

**DOI:** 10.7759/cureus.63065

**Published:** 2024-06-24

**Authors:** Yumi Furuya, Go Hirose, Takeo Hokari, Kenichi Hirose

**Affiliations:** 1 Rehabilitation Center, Hirose Hospital, Sagamihara, JPN; 2 Internal Medicine, Hirose Hospital, Sagamihara, JPN

**Keywords:** feeding problems, covid-19, dysphagia, lateral medullary syndrome, wallenberg syndrome

## Abstract

We experienced two consecutive cases of Wallenberg syndrome that developed after COVID-19 infection. Case 1 had symptoms such as a sore throat, but also complained of strong stress related to work and home at the same time. Therefore, it was initially thought due to a mental cause, but the psychiatrist denied depression and pointed out that it was a physical problem. After conducting neurological examinations and a videofluoroscopic (VF) swallowing examination, it was found to be dysphagia. Based on these findings, Wallenberg syndrome was diagnosed by MRI. Due to these events, it took a very long time to diagnose. Because of this experience, Case 2 was diagnosed with Wallenberg syndrome in a short time. Therefore, if symptoms like those in this case report such as feeding problems are observed after COVID-19 infection, it is considered meaningful to suspect Wallenberg syndrome from the beginning.

## Introduction

It is well-known that post-coronavirus disease (COVID)-19 syndromes not only cause respiratory diseases but also various pathological conditions [1]. Thrombosis is an important factor in the severity and mortality rate of COVID-19, as well as in brain fog [2,3]. Due to thrombosis, a high incidence of stroke associated with COVID-19 has also been reported. Indeed, it has been reported that approximately 1.4% of people infected with COVID-19 develop a stroke [4,5]. Wallenberg syndrome (lateral medullary syndrome) is a neurological disorder caused by damage to the outer part of the medulla oblongata [6]. The main cause is blockage of the vertebral artery (VA) and/or posterior inferior cerebellar artery (PICA), which supplies blood outside the medulla oblongata. The symptoms of Wallenberg syndrome include ipsilateral facial sensory disturbance on the affected side, contralateral trunk sensory disturbance, motor paralysis and sensory disturbance of the soft palate, pharynx, and larynx on the affected side, rotatory vertigo, ataxia, and Horner's syndrome. It is noteworthy that patients with Wallenberg syndrome often exhibit dysphagia and dysarthria due to bulbar palsy [7]. Brain magnetic resonance imaging (MRI) and magnetic resonance angiography (MRA) tests are performed to confirm the diagnosis [6]. A combination of drug therapy and rehabilitation is used as the treatment.

We report our experiences with two cases diagnosed as Wallenberg syndrome after developing dysphagia following COVID-19 infection. The diagnosis of Case 1 took a long time, but due to the experience with Case 1, Case 2 was diagnosed in a short time. Therefore, our experience with Case 1 was extremely helpful in diagnosing Case 2. It was considered useful to report these two cases that we experienced.

## Case presentation

Case 1

The patient involved was a 51-year-old female. Her blood pressure was normal. Her medical history included the presence of asthma. The patient had not been vaccinated. She developed a fever and tested positive for COVID-19 using a rapid test kit at the end of 2022 (day one). She experienced dizziness, unsteadiness, headache, sore throat, and high fever. The patient was treated at home, but even on day eight, her throat was still sore, and she was unable to eat or drink water. The patient was admitted to our hospital on day nine. On admission, she had swelling and pain in her mouth due to oral candidiasis, as well as sore throat and headache. As oral intake was difficult, even for water intake, she was receiving non-oral nutrition. Walking was also difficult because of disuse syndrome. In the head CT image taken at our hospital upon admission, there were no obvious findings. On the 16th day of hospitalization, the patient visited the otolaryngology department at Hospital A and was found to have left vocal cord paralysis, although swallowing dysfunction was denied. This patient complained of intense stress related to work and family at the time of onset, so she visited the psychiatry department on day 23. However, symptoms of depression were denied, and it was deemed necessary to conduct neurological and swallowing functional evaluations.

**Figure 1:**
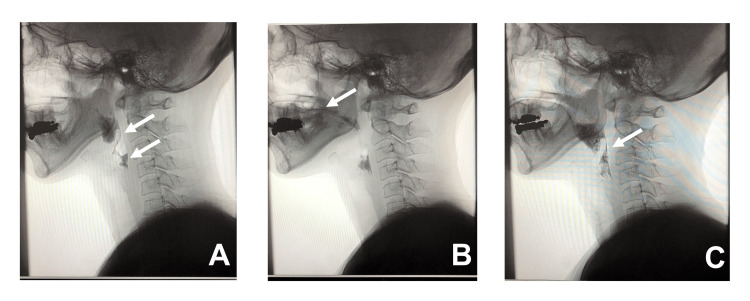
Videofluoroscopic swallowing (VF) examination images on day 31. (A) Although the swallowing reflex is evoked, pharyngeal contraction is weak, the inversion of the epiglottic is not achieved (upper arrow), and poor opening of the cricopharynx (lower arrow) is observed. (B) The bolus refluxes into the oral cavity (arrow). (C) Similar to A, the epiglottis did not invert (arrow), and the bolus did not pass through the pharynx.

A videofluoroscopic (VF) swallowing examination performed on day 31 is shown in Figure 1. Although a swallowing reflex was triggered, pharyngeal contraction was weak, and epiglottic inversion could not be achieved. The opening of the cricopharyngeal part was poor, and after refluxing into the mouth, swallowing was repeated with a similar reaction without any passage through the pharynx, indicating severe dysphagia. After receiving the results of the VF examination, we performed a neurological evaluation that revealed normal sensory/motor function of the face. However, the uvula was shifted to the right, and the curtain sign was observed. There was also only a slight contraction in response to touch stimulation of the posterior pharyngeal wall. In addition, the right side of the neck below the warmth pain sensation did not feel temperature when showering on the right side and did not know the sensation when the blood sugar measurement needle was attached to the right side. These findings led us to hypothesize the presence of Wallenberg syndrome. She was admitted to Hospital B for a definitive diagnosis on day 39. Head MRI revealed a high-signal subacute cerebral infarction focus in the left lateral medulla; thus, diagnosed with Wallenberg syndrome. Thereafter, the patient was transferred to a rehabilitation hospital, where she started oral intake; her functional oral intake scale (FOIS) improved to level 7; and returned to work six months after the onset of symptoms.

Case 2

An 80-year-old male was diagnosed with COVID-19 at the beginning of 2023 (day one). During treatment for COVID-19 at home, he suddenly vomited and fell and was transported to Hospital A on day 15. At Hospital A, he underwent a head MRI and was diagnosed with brainstem infarction (left lateral medulla) and was admitted to our hospital on the same day. At admission, the patient experienced vomiting, dysphagia, and dysarthria. His blood pressure was normal. His medical history included rheumatism, diabetes, and mild cerebral infarction without dysphagia (26 years ago). He had been vaccinated. From Case 1 experience, we suspected Wallenberg syndrome from the beginning and conducted a neurological evaluation. His neurological findings included mild left upper and lower extremity ataxia, left eyelid ptosis, drooping of the left corner of the mouth, tongue deviation to the left, insufficiency in soft palate elevation and curtain signs, and hoarseness. In addition, he experienced hypoesthesia to warmth and pain on the right side of his neck and below.

**Figure 2:**
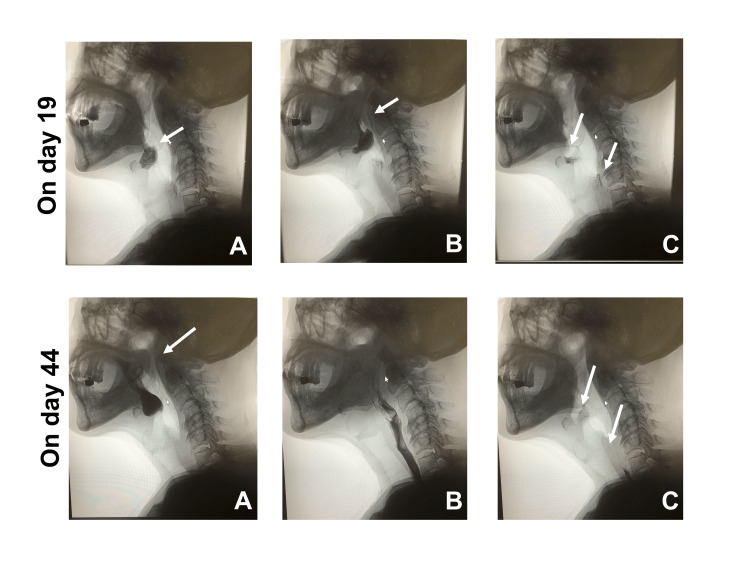
Videofluoroscopic swallowing (VF) examination images. The upper row shows VF performed on day 19 of illness. (A) Although the food bolus flows into the vallecula (arrow), the swallowing reflex is not evoked. (B) The soft palate elevates (arrow) with a delay, and the swallowing reflex is triggered. (C) However, residue was observed in the vallecula (upper arrow) and piriform recess (lower arrow). The lower row shows the VF examination performed on day 44 of illness. (A) Immediately after the bolus enters the vallecula, the soft palate elevates (arrow), triggering a swallowing reflex. (B) Good passage through the pharynx. (C) No residue is observed in the epiglottis (upper arrow) and piriform convexity (lower arrow).

**Figure 3:**
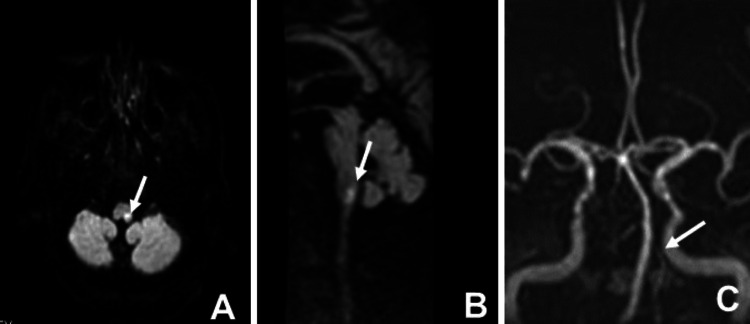
Brain magnetic resonance imaging (MRI) and magnetic resonance angiography (MRA) images. (A) Brain MRI shows a lesion in the left lateral medulla with hyperintensity on diffusion-weighted imaging (DWI) (arrow). (B) DWI brain sagittal view also showing a lesion in the lateral medulla with hyperintensity (arrow). (C) Brain MRA imaging showing the inability to extract the left VA (arrow).

VF examination performed on day 19 is shown in Figure 2. The bolus flowed into the vallecula but did not trigger a swallowing reflex; when it was finally triggered by a delay, there was a residue in the vallecula and pyriform sinus. These findings suggest that dysphagia was caused by bulbar palsy. Based on these findings, we re-viewed brain images taken at Hospital A on day 15 (Figure 3) and found a lesion in the left lateral medulla on MRI (DWI) images and an inability to extract the left VA on MRA. Therefore, the patient was diagnosed with Wallenberg syndrome.

For bulbar palsy symptoms, a training plan was established and interventions were made, with a speech therapist (ST) continuing oral function training while evaluating as appropriate. On day 21, oral intake was initiated with jelly while using enteral nutrition, and on day 23, the FOIS score was 3. After the second VF examination on day 26, a gradually advanced diet was administered. On day 37, the FOIS score had improved to 5. The third VF examination performed on day 44 is shown in Figure 2. The speed of triggering the swallowing reflex improved; there was no residue in the vallecula or pyriform sinus, and improvement in swallowing function was observed. On day 60, the FOIS score was 6, and he was discharged.

Table 1 shows the main symptoms of these two cases.

**Table 1:** Main symptoms. — indicates the absence of symptoms; + indicates the presence of symptoms; ++ indicates that the symptoms are severe.

Symptom	Case 1	Case 2
Loss of temperature and pain sensation on the same side of the face	—	—
Loss of temperature and pain sensation in the limbs on the opposite side	+	+
Diminished pharyngeal reflex	++	+
Deviation of the soft palate	+	+
Curtain sign	+	+
Atrophy and deviation of the tongue	—	+
Hoarseness	—	+
Ptosis	—	+

## Discussion

We have reported our experiences with two cases diagnosed as Wallenberg syndrome due to dysphagia following COVID-19 infection. Case 1 took time to diagnose Wallenberg syndrome. The reason for this was that the patient denied swallowing dysfunction when she visited the Otolaryngology Department of Hospital A on the 16th day of illness. Additionally, due to the patient’s unstable mental state, we considered the cause of the eating and swallowing disorder to be psychological, and it took time to verify each hypothesis, such as receiving a psychiatric diagnosis.

Based on the experience of Case 1, and also because we found two reports of cases where Wallenberg syndrome developed after COVID-19 infection [8,9], we suspected Wallenberg syndrome from the beginning in Case 2. In addition, we found one reported case of Wallenberg syndrome following vaccination against COVID-19 [10]. Thus, we were able to diagnose Case 2 in a short time. Therefore, our experience with Case 1 was extremely helpful in diagnosing Case 2. By the way, in both Cases 1 and Case2, loss of temperature and pain sensation on the same side of the face were absent. Regarding atrophy and deviation of the tongue, hoarseness, and ptosis, they were present in Case 1 but absent in Case 2 (Table 1).

The lesson learned from these two consecutive cases is that, among patients who complain of being unable to eat due to vomiting and headaches after COVID-19 infection, there may be those suffering from Wallenberg syndrome. Therefore, if symptoms like those in this case report such as feeding problems are observed after COVID-19 infection, it is considered meaningful to suspect Wallenberg syndrome from the beginning.

To diagnose Wallenberg syndrome, general diagnosis for COVID-19 infection, such as blood tests, is insufficient. Therefore, for patients with eating disorders, it is important to perform VF and/or videoendoscopic (VE) examination of swallowing early to assess swallowing function and identify dysphagia. If dysphagia is observed, an MRI examination must be performed to confirm the diagnosis of Wallenberg syndrome.

We encountered two cases of Wallenberg syndrome within a short period of one month at the end of 2022. During this period, 202 people were diagnosed as positive for COVID-19 at our hospital.

This period marked the peak of the eighth wave of COVID-19 in Japan. Regarding the eighth wave in Japan, by the end of 2022, the BA.5 strain accounted for approximately 70% of the infections, and the BQ.1 and BA.2.75 strains accounted for approximately 30% of the total infections [11]. This indicates an increased ability to escape from neutralizing antibodies induced by the B.1/2 virus or vaccine and an increased risk of incident. Because the climate was cold during this period, with many days when the minimum temperature was below freezing [12], it may be thought that the climate was conducive to stroke.

From these observations, it can be considered that the decreased immunity against SARS-CoV-2 and low temperatures may have promoted the onset of Wallenberg syndrome.

The typical post-infection symptoms of COVID-19 are wide-ranging including endothelial dysfunction [1]. This dysfunction is a pathological condition of inflammation and thrombosis induced by SARS-CoV-2 binding to the angiotensin-converting enzyme II (ACE-II) receptor expressed in vascular endothelial cells, depleting the ACE-II receptor, and relatively activating angiotensin II. Angiotensin II promotes vascular inflammation by inducing the production of cytokines and the expression of adhesion factors. Indeed, it has been reported that thrombosis is an important factor in the severity and mortality rate of COVID-19 [2,3]. The activation of matrix metalloproteinases due to the release of inflammatory cytokines, and the promotion of extracellular matrix degradation, leads to the weakening of the vascular wall. Therefore, there is a high possibility that arterial dissection may occur more easily.

Reflecting these reasons, a high incidence of stroke (approximately 1.4%) associated with COVID-19 has also been reported [4,5].

It is unclear whether the cause of these two cases was thrombus or vertebral artery dissection. However, the two cases of Wallenberg syndrome following COVID-19 infection that we reported hear are also likely to be directly caused by COVID-19 infection.

## Conclusions

We report our experiences with two cases diagnosed as Wallenberg syndrome due to dysphagia following COVID-19 infection. Case 1 had symptoms such as a sore throat and the patient was unable to even drink water. Initially, it was thought to be a mental issue as the patient was expressing strong stress related to work and home. However, a psychiatrist denied the symptoms of depression, and it was determined that a physical evaluation was necessary. After conducting tests such as a VF examination, dysphagia was revealed, and finally Wallenberg syndrome was diagnosed by MRI. Therefore, it took a very long time to reach a diagnosis. At admission, Case 2 experienced vomiting, dysphagia, and dysarthria. From Case 1's experience, we suspected Wallenberg syndrome from the beginning and conducted a neurological evaluation. For that reason, we were able to diagnose in a short time. Therefore, if symptoms like those in this case report are observed after COVID-19 infection, it is considered meaningful to suspect Wallenberg syndrome from the beginning.
